# Dietary chlorogenic acid modulates jejunal microbiota, short-chain fatty acids, and hypothalamic gene expression in broilers under high stocking density

**DOI:** 10.3389/fmicb.2026.1940516

**Published:** 2026-09-14

**Authors:** Dongying Bai, Yi Zhang, Yanli Wang, Fangshen Guo, Wenrui Zhen, Ziyue Zhao, Keyuan Lei, Chenjie Zhang, Cai Zhang, Yushu Zhang, Bingkun Zhang, Yanbo Ma

**Affiliations:** 1 Department of Animal Physiology, College of Animal Science and Technology, Henan University of Science and Technology, Luoyang, China; 2 Henan International Joint Laboratory of Animal Welfare and Health Breeding, College of Animal Science and Technology, Henan University of Science and Technology, Luoyang, China; 3 Innovative Research Team of Livestock Intelligent Breeding and Equipment, Science & Technology Innovation Center for Completed Set Equipment, Longmen Laboratory, Luoyang, China; 4 Department of Agriculture, Graduate School of Science and Technology, Shinshu University, Matsumoto, Japan; 5 State Key Laboratory of Animal Nutrition, Department of Animal Nutrition and Feed Science, College of Animal Science and Technology, China Agricultural University, Beijing, China

**Keywords:** broilers, chlorogenic acid, hypothalamus, jejunal microbiota, short-chain fatty acids, stocking density

## Abstract

High stocking density can impair broiler growth and is associated with alterations in intestinal microbial ecology and neuroendocrine regulation of feeding. Chlorogenic acid (CGA) has antioxidant, anti-inflammatory, and microbiota-modulating properties. This study evaluated dietary CGA in a 2 × 2 experimental arrangement defined by stocking density (normal vs. high) and CGA supplementation (0 vs. 1 g/kg). Two hundred and sixteen one-day-old male Arbor Acres (AA) broilers were assigned to ND (14 birds/m^2^), HD (22 birds/m^2^), NDCGA (14 birds/m^2^ + 1 g/kg CGA), or HDCGA (22 birds/m^2^ + 1 g/kg CGA), with six replicate pens per treatment. High stocking density was associated with poorer late-stage growth performance, age-dependent alterations in circulating cytokines, changes in jejunal microbial composition, lower concentrations of several jejunal short-chain fatty acids (SCFAs), and altered hypothalamic inflammatory- and appetite-related transcripts. Factorial analysis of growth-performance and serum-cytokine endpoints identified prominent stocking-density effects, whereas density × CGA interactions were limited to selected growth variables and were not consistently detected across outcomes. Accordingly, isolated HD–HDCGA pairwise differences were interpreted as treatment-combination differences unless supported by a significant interaction. Jejunal propionate and butyrate showed numerical upward trends in HDCGA compared with HD, but the pairwise differences were not significant. The transcriptional findings are consistent with possible involvement of AMPK/NF-κB- and MAPK-related regulation; however, direct pathway activation was not demonstrated. Collectively, these findings support coordinated associations among stocking density, dietary CGA, jejunal microbial ecology, local SCFA profiles, and hypothalamic transcription without establishing a causal microbiota–gut–brain signaling mechanism.

## 1 Introduction

The combined pressures of increasing demand for poultry products and intensive production have made stocking density an important management and welfare variable. High stocking density can reduce feeding opportunity and growth and can alter redox, immune, intestinal, behavioral, and neuroendocrine responses (Gržinić et al., 2023; Son et al., 2022; Sakr et al., 2025; Liu et al., 2023; Li et al., 2023). Because body mass increases rapidly during the production cycle, the physiological intensity of a fixed birds/m^2^ treatment also increases with age; therefore, birds/m^2^ describes the imposed allocation, whereas live-weight density provides useful biological context for the later growth period. European Union broiler-welfare legislation similarly defines stocking density by total live weight per usable area rather than by bird number alone (Council of the European Union, 2007); this regulatory framework is cited only as biological and welfare context and does not redefine the experimental treatments.

Nutritional interventions, including antioxidants, probiotics, phytogenic compounds, plant-derived bioactives, and other feed additives, can alleviate selected consequences of high stocking density, such as oxidative stress, inflammatory responses, and intestinal dysfunction. Recent independent work in high-density broilers also supports the capacity of diet-based interventions to modify oxidative, intestinal, and performance-related responses (Yang et al., 2025). These approaches should not be characterized as generally ineffective or unsustainable. Rather, the multifactorial nature of stocking-density stress makes compounds capable of influencing several connected physiological domains particularly relevant.

The microbiota–gut–brain axis provides a framework for examining associations among intestinal microbial communities, microbial metabolites, immune mediators, and central regulation of feeding. Although the cecum is the principal fermentative compartment in chickens, the jejunum is a major site of nutrient digestion and absorption and is directly exposed to dietary CGA. Jejunal microbiota and SCFA measurements therefore characterize a local small-intestinal ecological and metabolic environment rather than total intestinal fermentation or systemic metabolite exposure (Oakley et al., 2014; Shang et al., 2018; Du et al., 2024; Liu et al., 2021). Parallel cecal and circulating or portal SCFA measurements would be required to resolve those additional levels.

Chlorogenic acid (CGA) is a caffeoylquinic acid polyphenol with reported antioxidant, anti-inflammatory, and microbiota-modulating activities (Behne et al., 2023; Heitman and Ingram, 2017; Liu et al., 2024; Zhang B. et al., 2024; Tan et al., 2023). Previous studies from our group have shown that dietary CGA can influence growth, antioxidant and immune status, and intestinal endpoints in broilers exposed to high stocking density (Liu et al., 2023; Li et al., 2023; Bai et al., 2022). Consequently, the novelty of the present study is not the first demonstration that CGA can affect high-density-reared broilers. Instead, the present work integrates the four density × CGA treatment combinations with jejunal microbial ecology, local SCFA profiles, and hypothalamic inflammatory- and appetite-related transcription within the same experimental framework.

Selection of the hypothalamic targets was hypothesis-driven and was informed by prior evidence rather than by the present qRT-PCR results alone. In a previous hypothalamic RNA-seq study using the same 14 versus 22 birds/m^2^ high-stocking-density broiler platform, density-associated transcriptional changes were enriched in MAPK and Toll-like-receptor signaling and in biological processes related to inflammation and ingestive behavior; NPY, POMC, and GHRH were among the candidate feeding-related genes identified (Zhao et al., 2025). Together with evidence linking SCFA sensing and AMPK/NF-κB-related regulation to inflammatory responses, and prostaglandin signaling and MAPK-related processes to hypothalamic appetite regulation (Du et al., 2024; Liu et al., 2021; Hardie, 2013; Somade et al., 2019; Morton et al., 2014; Cao et al., 2021; Kim and Choi, 2010), these findings provided the rationale for prespecifying GPR43/AMPK/NF-κB/COX-2-related and EP3/MAPK/appetite-related transcript panels. These panels were intended as candidate regulatory modules and not as evidence that the complete signaling cascades were activated.

Accordingly, this study evaluated the effects of stocking density, dietary CGA supplementation, and their interaction on growth and circulating cytokine endpoints and examined the accompanying jejunal microbiota, local SCFA profiles, and hypothalamic transcriptional responses. The hypothalamic targets were assessed at the mRNA level. Because receptor activity, protein abundance, phosphorylation, neural-circuit activity, and circulating CGA or microbial metabolite exposure to the brain were not measured, the pathway-level interpretation was kept associative and hypothesis-supporting rather than causal.

## 2 Materials and methods

### 2.1 Animals and experimental design

A total of 216 healthy one-day-old male Arbor Acres (AA) broiler chicks were obtained from Hebei Huayu Poultry Breeding Co., Ltd. The feeding trial was conducted at the experimental facility of the College of Animal Science and Technology, Henan University of Science and Technology. All birds underwent health screening before housing. Birds were randomly allocated to four treatment combinations with six replicate pens per treatment: normal stocking density without CGA (ND), high stocking density without CGA (HD), normal stocking density with CGA (NDCGA), and high stocking density with CGA (HDCGA). Each replicate pen provided approximately 0.50 m^2^ usable floor area. ND and NDCGA pens housed 7 birds each, whereas HD and HDCGA pens housed 11 birds each, corresponding to nominal densities of 14 and 22 birds/m^2^, respectively. The 14 and 22 birds/m^2^ levels were selected to maintain continuity with the established high-density broiler platform used by our group, including previous CGA and related high-density studies (Bai et al., 2022; Zhang H. et al., 2024; Guo et al., 2025; Bai et al., 2026). The replicate pen was the independent experimental unit for treatment-level inference. Growth performance was recorded at pen level; for individual-animal endpoints, one sampled bird from each replicate pen provided the biological observation representing that pen.

Environmental conditions were regulated by an intelligent control system throughout the 42-day trial. Ambient temperature was maintained at 33 ± 1 °C during the first week and subsequently reduced by 1–2 °C per week until 25 ± 2 °C at trial completion; relative humidity was maintained at 60–70%. During days 0–7, the photoperiod was 24 L:0D at 20–30 lux. From day 8 onward, the photoperiod was adjusted to approximately 16–18 h light and 6–8 h dark at 5–10 lux. Beginning on day 12, a fixed 1-h dark period from 19:00 to 20:00 was included. Feed and water were available ad libitum. Routine facility health-management procedures were applied equally across all treatments; medication or vaccination status was not treated as an experimental factor, and no inference regarding an antibiotic-free production system was made.

Because birds gained substantial body mass during the trial, birds/m^2^ defined the randomized stocking-density factor and achieved biomass density was considered descriptively. Using the day-42 treatment mean body weights reported in Table 1 and the nominal bird densities, approximate terminal live-weight densities were 41.1, 60.0, 45.7, and 64.1 kg/m^2^ for ND, HD, NDCGA, and HDCGA, respectively. These treatment-level calculations are provided only to contextualize the increasing physiological intensity of crowding with age and are not presented as pen-level measured observations.

**Table 1:** Growth performance of broilers in the 2 × 2 factorial arrangement of stocking density and dietary CGA supplementation.

Parameter	Age/period	ND	HD	NDCGA	HDCGA	Pooled SEM	P-density	P-CGA	P-density × CGA
Body weight (g/bird)	7 d	134.21	132.47	135.03	133.26	2.27	0.102	0.441	0.989
	14 d	361.67	346.67	347.50	363.08	3.59	0.963	0.858	0.023
	21 d	807.17	752.83	815.33	772.33	16.00	0.052	0.563	0.812
	28 d	1480.00ᵃ	1051.17ᵇ	1464.83ᵃ	1452.83ᵃ	42.92	0.029	0.052	0.037
	35 d	2090.50ᵇ	1927.50ᵇ	2325.00ᵃ	2066.00ᵇ	39.11	0.017	0.033	0.562
	42 d	2937.33ᵇ	2725.67ᵇ	3266.33ᵃ	2915.33ᵇ	52.70	0.011	0.018	0.498
ADG (g/bird/day)	1–7 d	15.11	14.99	15.38	15.69	0.18	0.707	0.066	0.398
	8–14 d	25.74	26.66	27.20	27.53	0.40	0.282	0.053	0.608
	15–21 d	58.39	57.29	58.47	56.94	0.45	0.063	0.842	0.751
	22–28 d	82.08	79.15	83.90	80.15	1.05	0.046	0.380	0.797
	29–35 d	114.90ᵃᵇ	104.64ᵇ	117.07ᵃ	109.95ᵃᵇ	2.02	0.010	0.231	0.610
	36–42 d	129.58ᵃ	98.16ᵇ	126.47ᵃ	115.04ᵃ	3.59	0.003	0.295	0.134
ADFI (g/bird/day)	1–7 d	22.56	21.50	22.87	22.05	0.12	0.330	0.653	0.900
	8–14 d	39.32	39.85	38.83	39.60	0.30	0.308	0.559	0.849
	15–21 d	76.21	75.18	78.24	75.48	0.51	0.034	0.177	0.311
	22–28 d	126.63ᵃ	121.92ᵇ	128.70ᵃ	124.32ᵃᵇ	0.80	0.003	0.105	0.902
	29–35 d	172.40ᵃ	166.65ᵇ	176.89ᵃ	172.58ᵃ	1.13	0.015	0.012	0.707
	36–42 d	198.26ᵃᵇ	183.55ᶜ	200.15ᵃ	188.95ᵇᶜ	2.19	0.002	0.314	0.624
FCR (g/g)	1–7 d	1.49	1.51	1.47	1.44	0.18	0.910	0.317	0.575
	8–14 d	1.51	1.50	1.43	1.44	0.15	1.000	0.047	0.766
	15–21 d	1.31	1.31	1.34	1.33	0.01	0.780	0.172	0.780
	22–28 d	1.52	1.55	1.50	1.52	0.17	0.145	0.145	0.765
	29–35 d	1.51ᵃᵇ	1.65ᵃ	1.43ᵇ	1.59ᵃᵇ	0.31	0.010	0.197	0.851
	36–42 d	1.49ᵃᵇ	1.83ᵃ	1.39ᵇ	1.63ᵃ	0.42	0.003	0.096	0.567
	1–42 d‡	1.49	1.60	1.51	1.54	—	—	—	—

Values are treatment means from six replicate pens per treatment; pooled SEM is shown separately and was calculated from the common residual error term of the balanced factorial model. ND = normal density; HD = high density; NDCGA = normal density + 1 g/kg CGA; HDCGA = high density + 1 g/kg CGA. P-density, P-CGA, and P-density × CGA represent the main effect of stocking density, the main effect of CGA supplementation, and their interaction, respectively, from the 2 × 2 factorial ANOVA. Superscript letters indicate Tukey-adjusted *post hoc* comparisons among treatment combinations following the factorial ANOVA at *p* < 0.05. ‡ The days 1–42 FCR values are descriptive ratios calculated from the available phase-level treatment means; no pooled SEM or inferential p value was assigned to this derived row.

### 2.2 Diet composition and CGA supplementation

The basal diet was a pelleted corn–soybean meal-based diet formulated for starter (days 1–21) and grower (days 22–42) phases. Ingredient composition and calculated nutrient levels are presented in Table 2. CGA (purity ≥98%) was supplied by Changsha Sage Bio-tech Co., Ltd. (Changsha, China) and incorporated into the basal diet at 1 g/kg throughout the experimental period in the CGA-supplemented groups. The 1 g/kg inclusion level was selected on the basis of our previous high-stocking-density CGA study, in which this dietary level had been used in the same density model (Bai et al., 2022). CGA was administered through the complete diet rather than by individual oral gavage. The product is therefore described as a purified commercial CGA preparation. The product documentation available for this study did not specify whether the purified CGA was obtained by botanical extraction or chemical synthesis, and no unsupported manufacturing-route assignment is made.

**Table 2:** Ingredient composition and calculated nutrient levels of the basal diets.

Item	Starter (1–21 d)	Grower (22–42 d)
Ingredients, %		
Corn (CP, 7.9%)	52.790	57.780
Soybean meal (CP, 44.3%)	36.890	30.000
Corn gluten meal (CP, 62.5%)	0.000	2.430
Soybean oil	4.000	4.000
Sodium chloride	0.300	0.300
Choline chloride	0.300	0.260
Vitamin premix^1^	0.030	0.030
Trace element premix^2^	0.200	0.200
Limestone (stone powder)	1.222	1.171
Dicalcium phosphate	1.912	1.623
DL-Methionine	0.265	0.106
L-Lysine	0.038	0.045
Wheat bran	2.000	2.000
Calculated nutrient levels		
Metabolizable energy, MJ/kg	12.40	13.00
Crude protein, g/kg	211.8	198.4
Lysine, g/kg	11.4	10.5
Methionine, g/kg	4.9	4.8
Calcium, g/kg	10.2	8.5
Available phosphorus, g/kg	4.5	4.2
Total phosphorus, g/kg	6.9	6.3

^1^Vitamin premix supplied per kg of diet: vitamin A, 9,500 IU; vitamin D3, 62.5 μg; vitamin E, 30 IU; vitamin K3, 2.65 mg; vitamin B1, 2 mg; vitamin B6, 6 mg; vitamin B12, 0.025 mg; biotin, 0.0325 mg; folic acid, 1.25 mg; pantothenic acid, 12 mg; nicotinic acid, 50 mg. ^2^Trace element premix supplied per kg diet: Cu, 8 mg; Fe, 80 mg; Mn, 100 mg; Se, 0.15 mg; I, 0.35 mg. Minor deviations of the displayed ingredient percentages from 100% reflect rounding. Nutrient values are calculated formulation values, not newly analyzed values.

### 2.3 Growth performance measurement and sample collection

Feed intake was recorded throughout the experiment and body weight was recorded on days 7, 14, 21, 28, 35, and 42. Average daily feed intake (ADFI), average daily gain (ADG), body weight (BW), and feed conversion ratio (FCR) were calculated for the corresponding age intervals. An overall descriptive days 1–42 FCR was additionally calculated from the available phase-level treatment means as the ratio of cumulative mean feed intake to cumulative mean gain. Because pen-level cumulative feed-intake and gain observations were not retained for a formal overall-period analysis, this descriptive row was not assigned a pooled SEM or inferential *p*-value.

On days 21, 28, 35, and 42, one bird was sampled from each of the six replicate pens per treatment for blood collection by wing venipuncture (*n* = 6 replicate pens per treatment at each age). The sampling schedule was treated as cross-sectional by age; birds were not modeled as a longitudinal repeated panel across the four sampling ages. Blood was transferred into serum collection tubes and centrifuged at 3,000 × *g* for 10 min at 4 °C. Serum was aliquoted and stored at −20 °C pending analysis.

On day 42, one broiler was sampled from each of the six replicate pens per treatment (n = 6 replicate pens per treatment; 24 birds total) and euthanized by CO₂ inhalation. The day-42 sampled bird from each pen provided the hypothalamus and jejunal digesta used for the molecular, microbiome, and SCFA analyses. Hypothalamic tissues were collected immediately, dissected on ice, snap-frozen in liquid nitrogen, and stored at −80 °C. The gastrointestinal tract was excised, the jejunum was isolated aseptically, and jejunal digesta were transferred into sterile cryogenic tubes, immediately frozen in liquid nitrogen, and stored at −80 °C for subsequent microbiome and SCFA analyses. The sample-collection procedure for each bird was completed within approximately 15 min.

### 2.4 Determination of serum inflammatory cytokines

Serum concentrations of interleukin-1β (IL-1β), interleukin-6 (IL-6), tumor necrosis factor-α (TNF-α), and interleukin-4 (IL-4) were measured using commercially available enzyme-linked immunosorbent assay (ELISA) kits from Nanjing Jiancheng Bioengineering Institute (Nanjing, China) according to the manufacturer’s instructions.

Frozen serum samples were thawed at room temperature and centrifuged at 3,000 × g for 10 min to remove precipitates. Reagents were equilibrated to room temperature for 30 min and standards were prepared by serial dilution. Blank, zero-standard, standard, and sample wells were prepared as specified by the kit. Biotinylated detection antibody was added to all wells except blanks, followed by incubation at 37 °C for 30 min and five washing cycles. Streptavidin–horseradish peroxidase conjugate was then added, followed by a second 30-min incubation and washing step. Chromogenic substrates were added and the reaction was terminated after 10 min. Absorbance was measured at 450 nm. Standard curves were fitted using a four-parameter logistic model and sample concentrations were interpolated from the corresponding standard curves.

### 2.5 Reverse transcription quantitative real-time PCR (qRT-PCR)

Total RNA was extracted from homogenized hypothalamic tissue using TRIzol reagent (Invitrogen, United States). RNA concentration and purity were evaluated using a NanoDrop spectrophotometer (Thermo Fisher Scientific, United States), and samples with *A*₂₆₀/*A*₂₈₀ ratios between 1.8 and 2.0 were used for subsequent analyses. Following genomic-DNA removal, 1 μg total RNA was reverse-transcribed using an M-MLV Reverse Transcription Kit (AccurateBio, China). qRT-PCR was performed in a 20-μL reaction containing 10 μL 2 × SYBR Green Pro Taq HS Premix, 0.4 μL each of forward and reverse primer, 2 μL cDNA, and nuclease-free water.

The amplification program consisted of 95 °C for 30 s followed by 40 cycles of 95 °C for 10 s and 60 °C for 30 s. Reactions were performed in technical triplicate. GAPDH was used as the reference gene, consistent with the laboratory’s established broiler qPCR platform (Zhang H. et al., 2024; Guo et al., 2025; Bai et al., 2026), and relative expression was calculated using the 2^−^ΔΔCt method. Melting-curve analysis (65–95 °C) and amplification-efficiency testing were performed during the original qRT-PCR workflow as assay-quality checks. The retained revision archive does not contain the gene-specific numerical efficiency outputs or the original melting-curve plots; these results are therefore not reconstructed or estimated in the revised manuscript. Primer sequences and GenBank accession numbers are provided in Table 3.

**Table 3:** Primer sequences used for qRT-PCR and corresponding GenBank accession numbers.

Gene	Forward primer (5′–3′)	Reverse primer (5′–3′)	GenBank accession
GPR43	GGCTGTGGTCATCATGGTCAAC	GTGGTGAGGAGCAGAGCGTAG	NM_001318430.1
AMPK	CATCTGTCTCGCCCTCATCC	CCGTATCCCCAAATGCCACT	NM_001039603.2
IκBα	ACGGGGCAGATGTGAACAAAGTG	CCTGTATGCTGGCGTTGTCTCG	NM_001001472.3
NF-κB	TGCCCGAAGAGGTGCTGGAG	TGGAGGTGGCGAAGAATGAAACG	NM_001396395.1
IL-1β	ACTGGGCATCAAGGGCTA	GGTAGAAGATGAAGCGGGTC	NM_204524.2
IL-6	GCTGCGCTTCTACACAGA	TCCCGTTCTCATCCATCTTCTC	NM_204628.1
TNF-α	GAGCGTTGACTTGGCTGTC	AAGCAACAACCAGCTATGCAC	NM_204267.2
IL-10	AGAAATCCCTCCTCGCCAAT	AAATAGCGAACGGCCCTCA	NM_001004414.2
COX-2	CCGAATCGCAGCTGAATTCA	GAAAGGCCATGTTCCAGCAT	XM_040704521.2
mPGES-1	AGGCTCAGGAAGAAGGCATT	CACAGCTCCAAGGAAGAGGA	NM_001194983.1
EP3	GTGTAGGCAAAGGGGTTGGT	AGACCAAAAAGCCCCTCCAA	NM_001040468.2
JNK	TGAGCAGAAGCAAGCGTGACAAC	CGCTCAAGGATGGCATCATAGGC	XM_040703166.1
p38	GCAGAACAGTGGCGTTATCC	TCCCCTCTTTCCATCCATGTC	XM_001232615.5
ERK	GCTTCATCGCGACCTCAAAC	GAGCCAGTCCGAAGTCACAA	NM_204150.2
NPY	CCTTCGATGTGGTGATGGGA	ATGCACTGGGAATGACGCTA	NM_205473.2
GHRH	TGATGGACAGCCGTTACCAC	GCTGGGAAACCCCTCTAACC	XM_015296359.4
POMC	GAGAACAGCAAGTGCCAGGA	ACGTACTTGCGGATGCTCTC	NM_001398117.1
SST	TCTCAGAGCCAAGCCAGACAG	CCAGCTCCAGTCTCACTTCA	NM_205336.1
GAPDH	TGCTGCCCAGAACATCATCC	ACGGCAGGTCAGGTCAACAA	NM_204305.2

GAPDH was used as the reference transcript. Melting-curve analysis and amplification-efficiency testing were performed during the original qRT-PCR workflow; the gene-specific numerical efficiency outputs and original melting-curve plots are not retained in the current revision archive and are not reconstructed.

### 2.6 16S rRNA gene amplicon sequencing and bioinformatic analysis

Microbial genomic DNA was extracted from jejunal digesta using the cetyltrimethylammonium bromide method and evaluated on 1% agarose gels. DNA was diluted to 1 ng/μL before amplification. The V3–V4 region of the bacterial 16S rRNA gene was amplified using primers 341F (5′-CCTAYGGGRBGCASCAG-3′) and 806R (5′-GGACTACNNGGGTATCTAAT-3′). Each PCR was performed in a 30-μL reaction containing 15 μL of 2 × Phusion High-Fidelity PCR Master Mix with GC Buffer (New England Biolabs, United States), 1 μL each of the forward and reverse primers, 10 μL template DNA containing approximately 10 ng DNA, and 3 μL nuclease-free water. Thermal cycling consisted of 98 °C for 1 min; 30 cycles of 98 °C for 10 s, 50 °C for 30 s, and 72 °C for 30 s; and a final extension at 72 °C for 5 min. Amplicons were pooled at equimolar ratios, separated on a 2% agarose gel, and purified using a Universal DNA Purification Kit (Tiangen, China). Sequencing libraries were constructed using the NEBNext Ultra DNA Library Prep Kit for Illumina, evaluated using an Agilent 5,400 system, quantified by qPCR, and sequenced using paired-end 2 × 250-bp reads on an Illumina NovaSeq 6,000 platform by Novogene Co., Ltd. (Beijing, China).

Raw reads were processed in QIIME 2 (version 2019.1). Demultiplexing, quality filtering, denoising, merging, and chimera removal were conducted using DADA2 to generate amplicon sequence variants (ASVs). Taxonomic classification was performed using the QIIME 2 feature-classifier plugin against the Greengenes 13_8 reference database at 99% sequence similarity, with the reference sequences restricted to the V3–V4 region; chloroplast and mitochondrial sequences were removed before downstream analyses. Sequencing-depth sufficiency was examined with rarefaction curves for Faith’s phylogenetic diversity and observed ASVs (Figures 1A,B). The between-group alpha-diversity comparison used Chao1, Shannon, and Simpson indices (Figure 1C), and beta-diversity was visualized by principal coordinates analysis (PCoA) based on Bray–Curtis dissimilarity (Figure 1E). Taxonomic profiles and linear discriminant analysis effect size (LEfSe) were used to characterize treatment-associated taxa. The original bioinformatic workflow specified default parameters unless otherwise stated; accordingly, the LEfSe analysis used the default Kruskal–Wallis alpha of 0.05 and logarithmic LDA score threshold of 2.0. Within-group co-occurrence networks were constructed from Spearman correlations among major taxa, and exploratory genus-level correlations with growth and cytokine variables were also assessed by Spearman correlation. Network and phenotype-correlation results were interpreted as associative rather than causal.

**Figure 1:**
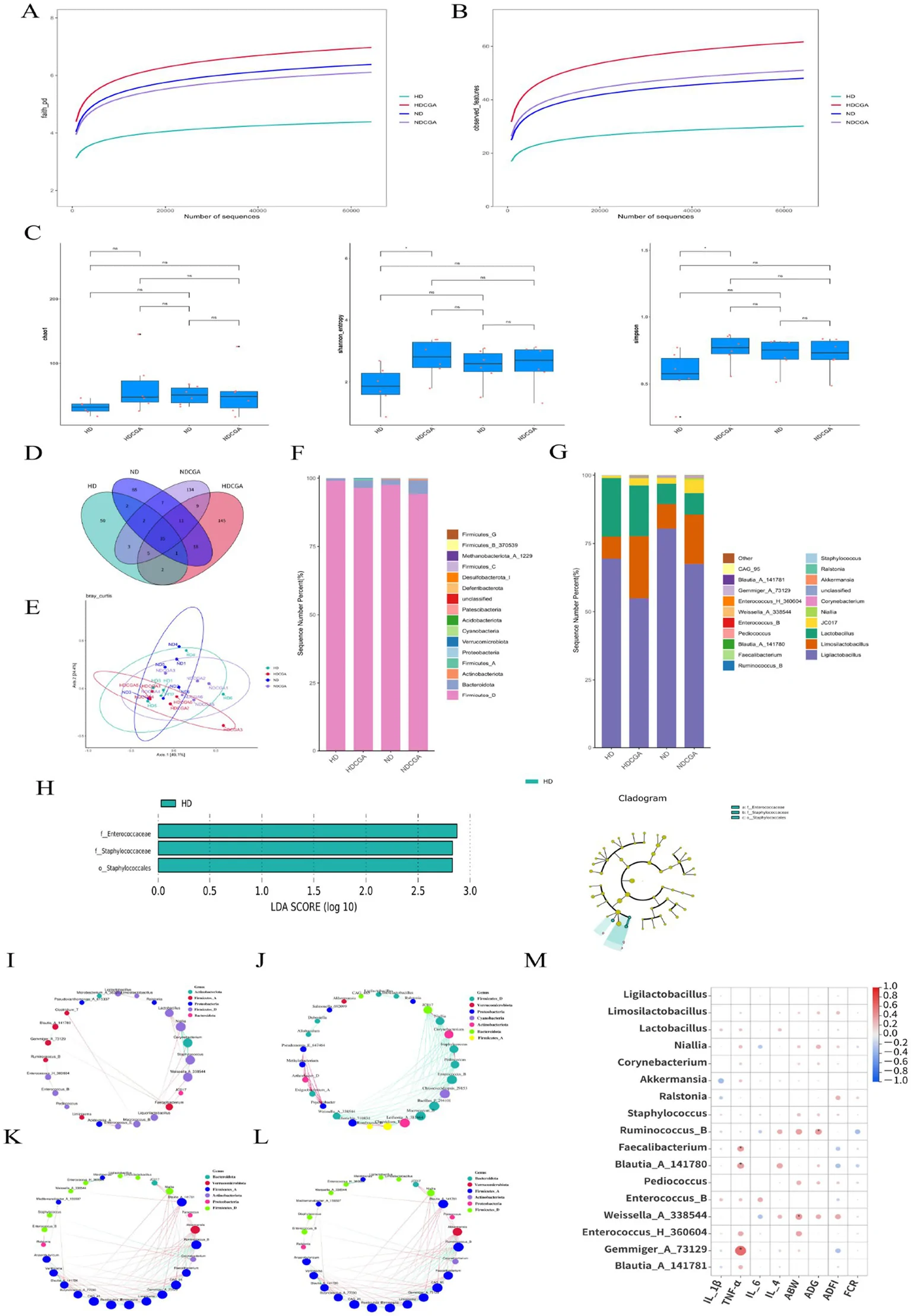
Effects of stocking density and dietary CGA supplementation on jejunal microbial ecology at day 42. Panels **(A,B)** show rarefaction curves for Faith’s phylogenetic diversity and observed ASVs, respectively. Panel **(C)** shows Chao1, Shannon, and Simpson alpha-diversity indices. Panel **(D)** shows ASV overlap. Panel **(E)** shows Bray–Curtis PCoA. Panels **(F,G)** show taxonomic composition at the phylum and genus levels, respectively. Panel **(H)** shows LEfSe output using the default Kruskal–Wallis alpha of 0.05 and logarithmic LDA score threshold of 2.0. Panels **(I–L)** show the exploratory co-occurrence networks for ND, HD, NDCGA, and HDCGA, respectively, and **(M)** shows exploratory genus-level correlations with growth and cytokine variables. In **(I–L)**, node size represents relative taxon abundance and red and green edges indicate positive and negative correlations, respectively. In **(M)**, red and blue indicate positive and negative correlations, respectively. ASV, amplicon sequence variant. Significance symbols are defined as *p < 0.05, **p < 0.01, and ***p < 0.001 where applicable. Ordination, taxonomic enrichment, co-occurrence, and correlation patterns are interpreted as descriptive or associative evidence rather than proof of causal ecological or host effects.

### 2.7 SCFA analysis

Acetic, propionic, butyric, isobutyric, valeric, isovaleric, and hexanoic acid standards were prepared at 100 mg/mL and serially diluted. A 4-methylvaleric acid solution in diethyl ether (375 μg/mL) was used as the internal standard. A 10-point calibration series (0.02–500 μg/mL) was prepared for each SCFA. Fresh working solutions were prepared for each analytical run.

A known mass of jejunal digesta was homogenized with 500 μL deionized water and glass beads and centrifuged at 12,000 × g for 10 min at 4 °C. A 200-μL aliquot of supernatant was mixed with 100 μL 15% phosphoric acid, 20 μL internal standard, and 280 μL diethyl ether, vortexed, and centrifuged. The upper organic phase was collected and filtered through a 0.22-μm membrane. SCFAs were quantified using a Shimadzu GC-2014 ATF gas chromatograph equipped with a 30 m × 0.25 mm × 0.5 μm capillary column. Nitrogen was used as carrier gas; injector and detector temperatures were 180 °C, and the column temperature was increased from 80 to 170 °C at 5 °C/min. Concentrations are reported as μg/g wet jejunal digesta.

### 2.8 Statistical analysis

Growth-performance, serum-cytokine, jejunal SCFA, and qRT-PCR outcomes were analyzed according to the 2 × 2 factorial arrangement by two-way ANOVA, with stocking density (normal vs. high), dietary CGA supplementation (0 vs. 1 g/kg), and the stocking density × CGA interaction included in the model. The replicate pen was the independent experimental unit. Growth-performance variables were recorded at pen level. For serum cytokines, one bird sampled from each replicate pen provided one biological observation representing that pen at each age (*n* = 6 replicate pens per treatment), and each age was analyzed separately as a cross-sectional endpoint rather than as a repeated within-bird panel. For the day-42 SCFA and qRT-PCR endpoints, one sampled bird from each replicate pen provided one biological observation representing that pen (*n* = 6 replicate pens per treatment). When comparisons among the four treatment combinations were displayed, Tukey-adjusted *post hoc* contrasts were obtained from the same factorial model; no separate four-group omnibus model was retained.

The factorial *p*-values for stocking density, CGA supplementation, and their interaction are reported in Tables 1, 4 and are the primary inferential results. Figures 2–4 were likewise analyzed using the 2 × 2 two-way ANOVA framework, with Tukey-adjusted *post hoc* comparisons used for the displayed treatment-combination lettering. Microbiome-specific analyses (rarefaction, Bray–Curtis PCoA, LEfSe, co-occurrence networks, and Spearman correlations) were conducted as described in Section 2.6 because these procedures address ecological structure and feature discovery rather than conventional factorial mean comparisons. IBM SPSS Statistics version 26.0 was used for factorial analyses, GraphPad Prism 9.0 was used for qRT-PCR graphics, and *p* < 0.05 was considered statistically significant.

**Table 4:** Serum cytokine concentrations in the 2 × 2 factorial arrangement of stocking density and dietary CGA supplementation.

Parameter	Age/period	ND	HD	NDCGA	HDCGA	Pooled SEM	P-density	P-CGA	P-density × CGA
IL-1β (ng/mL)	21 d	21.39	26.10	24.37	24.68	0.87	0.054	0.532	0.088
	28 d	36.23ᵇᶜ	53.31ᵃ	27.63ᶜ	39.75ᵇ	2.79	0.012	0.048	0.643
	35 d	32.74	37.05	32.14	35.08	1.56	0.095	0.542	0.744
	42 d	27.40ᵇ	33.17ᵃ	24.64ᵇ	29.32ᵃᵇ	1.02	0.004	0.054	0.739
IL-6 (ng/mL)	21 d	47.05ᵇ	62.41ᵃ	44.60ᵇ	55.82ᵃᵇ	2.64	0.008	0.326	0.649
	28 d	54.40	57.14	53.45	54.94	2.72	0.353	0.487	0.782
	35 d	38.17ᵇ	52.06ᵃ	37.55ᵇ	39.27ᵇ	2.20	0.060	0.103	0.136
	42 d	36.58ᵇ	52.80ᵃ	34.84ᵇ	42.87ᵃᵇ	2.69	0.013	0.204	0.368
TNF-α (ng/mL)	21 d	22.52	24.15	19.95	24.02	0.71	0.043	0.318	0.365
	28 d	25.86ᵃᵇ	29.43ᵃ	23.17ᵇ	27.11ᵃᵇ	0.89	0.009	0.066	0.887
	35 d	21.38ᵇ	30.84ᵃ	21.22ᵇ	23.38ᵇ	1.31	0.024	0.124	0.139
	42 d	27.74	29.57	25.11	28.49	0.81	0.039	0.131	0.518
IL-4 (ng/mL)	21 d	26.76	23.79	28.92	24.69	0.99	0.025	0.314	0.676
	28 d	36.10ᵃ	29.61ᵇ	45.76ᵃ	30.22ᵇ	2.23	0.007	0.180	0.235
	35 d	41.68	37.57	42.64	41.50	2.93	0.367	0.401	0.608
	42 d	38.59ᵃ	23.52ᵇ	38.76ᵃ	36.84ᵃ	2.47	0.038	0.094	0.102

Values are treatment means; pooled SEM is shown separately and was calculated from the common residual error term of the balanced factorial model. Each sampling age was analyzed as a separate cross-sectional endpoint, with one sampled bird from each of the six replicate pens per treatment providing one observation representing that pen. P-density, P-CGA, and P-density × CGA represent the main effect of stocking density, the main effect of CGA supplementation, and their interaction, respectively, from the 2 × 2 factorial ANOVA. Superscript letters indicate Tukey-adjusted *post hoc* comparisons among treatment combinations following the factorial ANOVA at *p* < 0.05.

**Figure 2:**
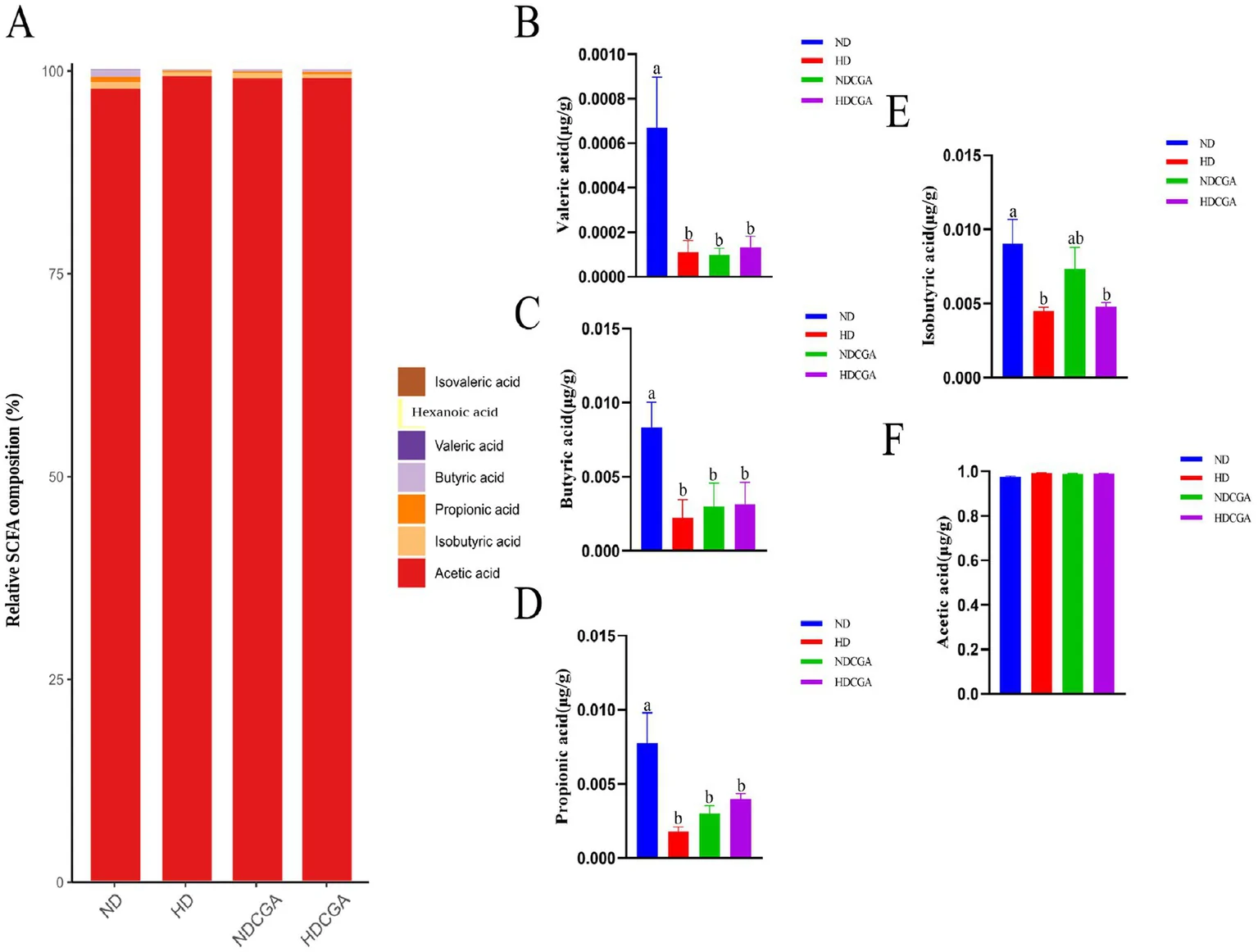
Jejunal short-chain fatty acid concentrations at day 42. Panel **(A)** shows the relative composition of measured SCFAs (%), and Panels **(B–F)** show valerate, butyrate, propionate, isobutyrate, and acetate concentrations, respectively, reported as μg/g wet jejunal digesta. For these endpoints, one bird sampled from each replicate pen provided one observation representing that pen (*n* = 6 replicate pens per treatment). The panel **(A)** axis and legend use SCFA terminology, including hexanoic acid. SCFA endpoints were analyzed by two-way ANOVA with stocking density, CGA supplementation, and their interaction, followed by Tukey-adjusted *post hoc* comparisons. Bars bearing different lowercase letters differ in these *post hoc* comparisons (*p* < 0.05); bars sharing a letter are not significantly different. No individual SCFA differed significantly between HD and HDCGA; propionate and butyrate showed numerical upward trends only. ND, normal density; HD, high density; NDCGA, normal density + 1 g/kg CGA; HDCGA, high density + 1 g/kg CGA.

**Figure 3:**
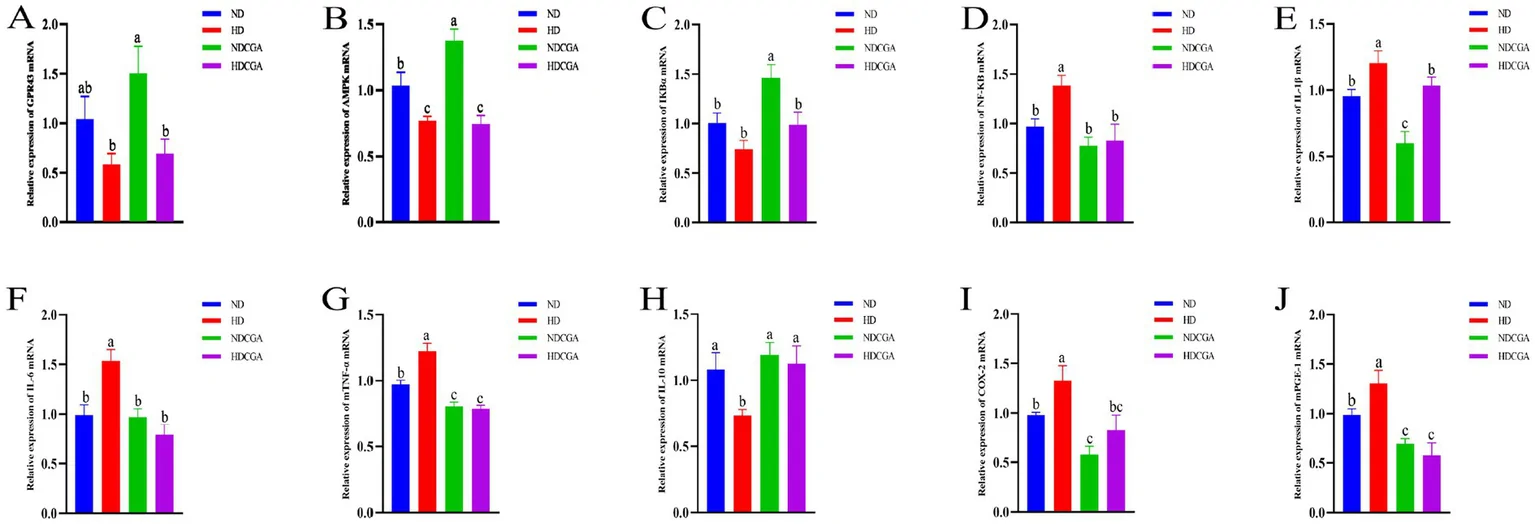
Relative hypothalamic expression of GPR43/AMPK/NF-κB/COX-2-related genes at day 42: **(A)** GPR43, **(B)** AMPK, **(C)** IκBα, **(D)** NF-κB, **(E)** IL-1β, **(F)** IL-6, **(G)** TNF-α, **(H)** IL-10, **(I)** COX-2, and **(J)** mPGES-1. GAPDH was used for normalization. One bird sampled from each replicate pen provided one observation representing that pen (*n* = 6 replicate pens per treatment). Each endpoint was analyzed by two-way ANOVA with stocking density, CGA supplementation, and their interaction, followed by Tukey-adjusted *post hoc* comparisons. Bars bearing different lowercase letters differ in these *post hoc* comparisons (*p* < 0.05); bars sharing a letter are not significantly different. The measurements represent mRNA abundance and do not demonstrate receptor activation, protein abundance, phosphorylation, nuclear translocation, or pathway flux.

**Figure 4:**
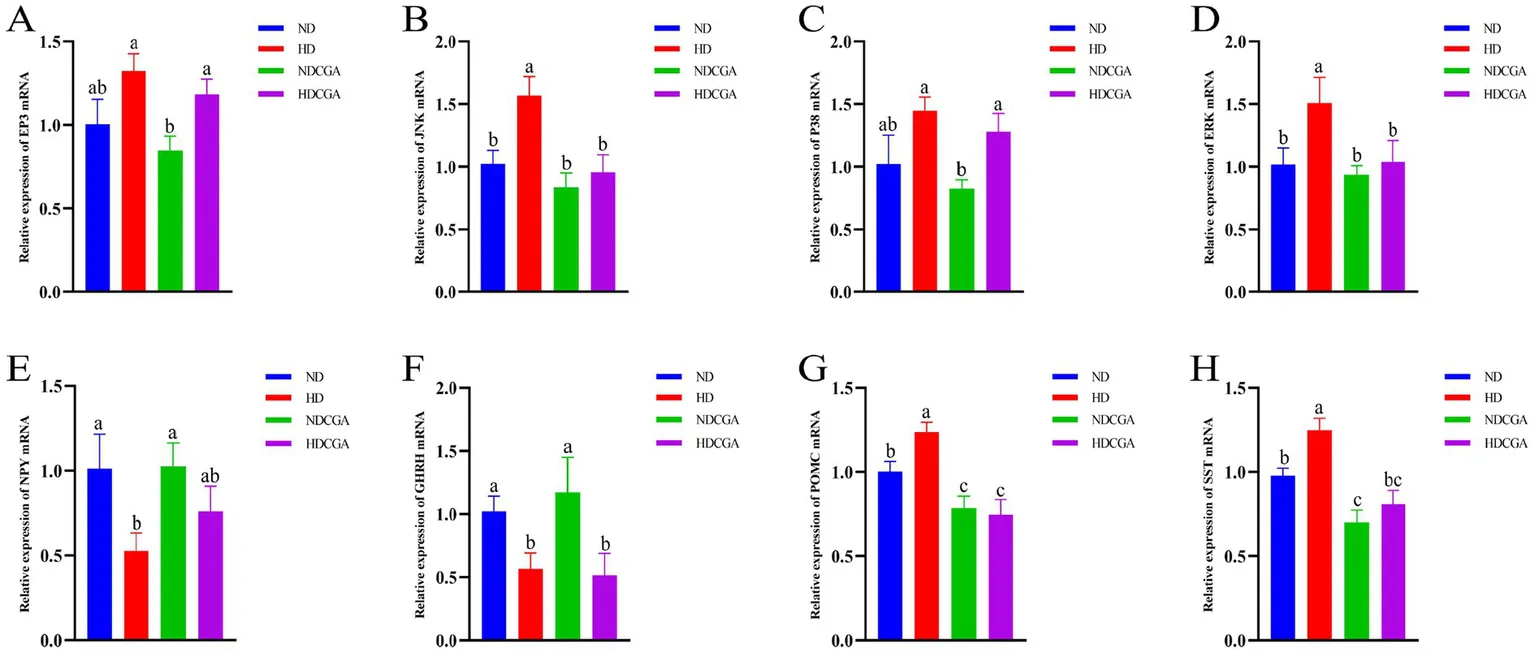
Relative hypothalamic expression of EP3/MAPK- and appetite-related genes at day 42: **(A)** EP3, **(B)** JNK, **(C)** p38, **(D)** ERK, **(E)** NPY, **(F)** GHRH, **(G)** POMC, and **(H)** SST. One bird sampled from each replicate pen provided one observation representing that pen (*n* = 6 replicate pens per treatment). Each endpoint was analyzed by two-way ANOVA with stocking density, CGA supplementation, and their interaction, followed by Tukey-adjusted *post hoc* comparisons. Bars bearing different lowercase letters differ in these *post hoc* comparisons (*p* < 0.05); bars sharing a letter are not significantly different. EP3 was not significantly different between HD and HDCGA. JNK and ERK results refer to transcript abundance; MAPK phosphorylation was not measured.

## 3 Results

### 3.1 Effects of stocking density and CGA on growth performance

Factorial analysis showed significant main effects of stocking density on BW at days 28, 35, and 42; ADG during days 22–28, 29–35, and 36–42; ADFI during days 15–21, 22–28, 29–35, and 36–42; and FCR during days 29–35 and 36–42 (P-density < 0.05; Table 1). Significant main effects of CGA were detected for BW at days 35 and 42, ADFI during days 29–35, and FCR during days 8–14 (P-CGA < 0.05). Significant stocking density × CGA interactions were detected for BW at days 14 and 28 (P-density × CGA < 0.05), whereas the remaining interaction terms were not significant.

Tukey-adjusted *post hoc* comparisons from the factorial model were used to facilitate interpretation of the four treatment combinations. HDCGA had higher day-28 BW and higher ADG during days 36–42 than HD. Because most later growth endpoints did not show a significant density × CGA interaction, isolated HD–HDCGA pairwise differences were not interpreted as evidence of a uniform density-specific CGA rescue. The descriptive days 1–42 FCR was approximately 1.49, 1.60, 1.51, and 1.54 for ND, HD, NDCGA, and HDCGA, respectively; this summary value was not subjected to inferential testing.

### 3.2 Age-dependent serum cytokine responses

Serum cytokine concentrations are shown in Table 4. Factorial analysis identified significant main effects of stocking density for IL-1β at days 28 and 42; IL-6 at days 21 and 42; TNF-α at days 21, 28, 35, and 42; and IL-4 at days 21, 28, and 42 (P-density < 0.05). A significant main effect of CGA was detected for IL-1β at day 28 (P-CGA < 0.05). No stocking density × CGA interaction reached statistical significance for the serum cytokine endpoints at any sampling age (P-density × CGA > 0.05).

Tukey-adjusted *post hoc* comparisons following the factorial model showed lower IL-1β in HDCGA than HD at day 28, lower IL-6 and TNF-α at day 35, and higher IL-4 at day 42. These findings are therefore described as age-specific treatment-associated differences. Because the corresponding interaction terms were not significant, they are not interpreted as evidence of a uniform density-specific anti-inflammatory effect of CGA across days 21–42.

### 3.3 Jejunal microbiota

Rarefaction curves for Faith’s phylogenetic diversity and observed ASVs approached a plateau as sequencing depth increased (Figures 1A,B), and Good’s coverage was approximately 99.8%, supporting adequate sequencing depth for the included samples. In the alpha-diversity comparison (Figure 1C), Chao1, Shannon, and Simpson indices were displayed; Shannon and Simpson were higher in HDCGA than HD in the between-group comparison (*p* < 0.05), whereas Chao1 did not show a corresponding significant difference. The Venn diagram showed 50, 88, 134, and 145 unique ASVs in HD, ND, NDCGA, and HDCGA, respectively (Figure 1D). In the Bray–Curtis PCoA ordination, HD and ND occupied partly distinct regions of the plot, while HDCGA was positioned closer to the normal-density clusters (Figure 1E). This ordination pattern is described visually and is not interpreted as a formal test of group separation.

At the phylum and genus levels, between-group microbiome comparisons indicated lower Bacteroidota, Lactobacillus, and Akkermansia and higher Staphylococcus in HD relative to ND, with changes in the opposite direction in HDCGA relative to HD (*p* < 0.05; Figures 1F,G). LEfSe identified enrichment of Enterococcaceae, Staphylococcaceae, and Staphylococcales in HD (Figure 1H). Genus-level correlation analysis identified associations between selected taxa and growth or cytokine variables (Figures 1I–M). These taxonomic and correlation findings are presented as associations; they do not establish pathogenic activity, cross-feeding, ecological competition, or causal effects on host phenotypes.

### 3.4 Jejunal SCFA concentrations

The jejunal SCFA profile at day 42 is shown in Figure 2. Acetate was the most abundant measured SCFA. Compared with ND, HD had lower concentrations of valerate, propionate, butyrate, and isobutyrate in Tukey-adjusted *post hoc* comparisons following the factorial model (*p* < 0.05). No individual SCFA differed significantly between HD and HDCGA. Propionate and butyrate were numerically higher in HDCGA than HD, but these differences did not reach statistical significance (*p* > 0.05). Therefore, the data do not support a claim that CGA restored jejunal SCFA concentrations.

### 3.5 Hypothalamic GPR43/AMPK/NF-κB/COX-2-related transcription

Within the day-42 factorial analysis, HD showed lower AMPK mRNA abundance and higher NF-κB, IL-1β, IL-6, TNF-α, COX-2, and mPGES-1 mRNA abundance than ND, together with lower IL-10 expression in Tukey-adjusted *post hoc* comparisons following the factorial model (*p* < 0.05; Figure 3). GPR43 mRNA showed a numerical decrease in HD but did not differ significantly from ND. Compared with HD, HDCGA showed lower abundance of several NF-κB-related inflammatory transcripts and higher IL-10 expression (*p* < 0.05 in Tukey-adjusted *post hoc* comparisons following the factorial model). These measurements demonstrate treatment-associated transcriptional differences but do not establish GPR43 activation, AMPK phosphorylation, NF-κB nuclear translocation, or pathway flux.

### 3.6 Hypothalamic EP3/MAPK- and appetite-related transcription

Within the day-42 factorial analysis, HD showed higher JNK and ERK mRNA abundance than ND, whereas EP3 and p38 showed non-significant numerical increases (Figure 4). NPY and GHRH mRNA abundance was lower and POMC and SST abundance was higher in HD than ND (*p* < 0.05 in Tukey-adjusted *post hoc* comparisons following the factorial model). Compared with HD, HDCGA showed lower JNK, ERK, POMC, and SST expression; NPY showed a non-significant upward trend, and EP3 did not differ significantly. These data are therefore interpreted as MAPK- and appetite-related transcriptional patterns rather than evidence that CGA directly inhibited an EP3-initiated signaling cascade.

## 4 Discussion

The present study examined whether dietary CGA modifies growth, jejunal microbial, local SCFA, and hypothalamic transcriptional responses associated with high stocking density. For growth-performance and serum-cytokine endpoints, interpretation is guided primarily by the factorial main effects and stocking density × CGA interactions, while Tukey-adjusted contrasts from the same factorial model provide complementary treatment-combination interpretation. Transcript-level and correlation-based findings are interpreted separately from direct pathway activation or causal microbiota–brain signaling.

### 4.1 Growth performance and stocking-density context

The factorial analysis supports a pronounced contribution of stocking density to late-stage broiler growth, with significant density main effects on several BW, ADG, ADFI, and FCR endpoints as birds approached market age. Significant CGA main effects were also detected for selected BW, ADFI, and FCR endpoints, whereas density × CGA interactions were limited to BW at days 14 and 28. This pattern is biologically compatible with the increasing biomass load per unit floor area as birds gained weight. Independent studies likewise report that increasing stocking density can depress growth and alter stress-related outcomes in broilers, particularly during the finisher period (Son et al., 2022; Sakr et al., 2025; Dozier et al., 2005; Dozier et al., 2006; Yang et al., 2025). The approximate day-42 live-weight densities calculated from the treatment means in Table 1 were substantially higher in the high-density groups than in the corresponding normal-density groups; these values are presented only as biological context for the imposed stocking-density treatment.

The limited number of significant interaction terms is important for interpreting CGA responsiveness under different stocking densities. An HD–HDCGA pairwise difference can identify a biologically relevant treatment-combination contrast, but it does not by itself establish that the CGA effect is specifically modified by stocking density when the formal interaction is not significant. Accordingly, density-specific claims are restricted to endpoints supported by the factorial interaction, while other pairwise differences are reported as treatment-combination effects. This distinction keeps the biological interpretation aligned with the statistical design (Liu et al., 2023; Li et al., 2023; Bai et al., 2022).

### 4.2 Circulating cytokine profile

High stocking density was associated with an age-dependent circulating cytokine profile. The factorial analysis identified density main effects for several cytokines at selected ages, but no significant density × CGA interaction was detected for any serum cytokine endpoint. Different cytokines differed at different ages, and not all pro-inflammatory markers remained elevated across the entire sampling period. This temporal heterogeneity is compatible with broader reports that stocking-density stress responses vary with age, environmental context, and measured endpoint (Son et al., 2022; Sakr et al., 2025; Yang et al., 2025). These data do not support a description of a sustained systemic inflammatory state; instead, they indicate temporal heterogeneity and support interpreting circulating cytokines as systemic biomarkers rather than histological evidence of tissue inflammation.

Several HD–HDCGA differences were observed at individual ages, including lower IL-1β, IL-6, or TNF-α and higher IL-4 in HDCGA at selected time points. These findings are compatible with treatment-associated immunomodulatory responses; however, because the density × CGA interaction terms were not significant, they do not demonstrate a uniform density-specific anti-inflammatory effect of CGA. The distinction is important because cytokine concentrations can be influenced by age, growth, nutritional state, and transient immune activity in addition to stocking density.

### 4.3 Jejunal microbiota and local SCFAs

The jejunum was selected to characterize the local microbial and metabolite environment at a major site of nutrient digestion and absorption and direct dietary exposure. This differs from the cecum, where microbial biomass and fermentative activity are substantially greater (Oakley et al., 2014; Shang et al., 2018). The present jejunal SCFA values should therefore be interpreted as local concentrations determined by the balance among microbial production, digesta transit, and host absorption, not as total intestinal fermentation or circulating exposure (Du et al., 2024; Liu et al., 2021). The absence of paired cecal and plasma or portal SCFA measurements is a limitation.

The between-group microbiome comparisons indicated density- and CGA-associated shifts in Bacteroidota, Lactobacillus, Akkermansia, Staphylococcus, and other taxa. These observations support remodeling of the jejunal community but do not establish the function of individual taxa. Independent literature supports biologically plausible roles for lactobacilli and Akkermansia in intestinal ecological stability (Djukovic et al., 2022; Liu et al., 2022), and dietary CGA has been associated with microbiota shifts in other animal models (Chen et al., 2019). Nevertheless, correlation networks cannot prove cross-feeding, competitive exclusion, pH-mediated niche changes, or direct effects on host growth. Interpretation is therefore restricted to the measured taxonomic and correlation patterns.

The SCFA findings require similar restraint. HD had lower concentrations of several individual jejunal SCFAs relative to ND, but no individual SCFA differed significantly between HD and HDCGA. The numerical upward trends in propionate and butyrate after CGA supplementation are directionally consistent with a change in the local metabolic environment, but they do not demonstrate restoration of SCFA production or mediation of the hypothalamic response (Du et al., 2024; Liu et al., 2021). Establishing such a chain would require microbial functional measurements together with cecal, portal, and/or systemic metabolite data.

### 4.4 Hypothalamic transcription and mechanistic scope

The hypothalamic findings show coordinated changes in inflammatory- and appetite-related transcripts. HD was associated with lower AMPK expression and higher abundance of several NF-κB-related transcripts, while HDCGA showed lower abundance of several of these genes in the treatment-combination comparison. AMPK and NF-κB are biologically plausible regulatory nodes (Hardie, 2013; Somade et al., 2019); however, GPR43 itself was not significantly reduced by HD, and the study did not quantify GPR43 activity, AMPK phosphorylation, NF-κB nuclear translocation, protein abundance, or functional pathway responses. The most defensible conclusion is therefore that the transcriptional pattern is consistent with possible AMPK/NF-κB-related regulation rather than direct activation of a GPR43/AMPK pathway by CGA.

The same evidence hierarchy applies to the EP3/MAPK interpretation. JNK and ERK transcripts were elevated in HD and lower in HDCGA, whereas EP3 was not significantly altered and MAPK phosphorylation was not measured. EP receptors, MAPK signaling, and hypothalamic appetite regulation provide biological context for the candidate pathway (Morton et al., 2014; Cao et al., 2021; Kim and Choi, 2010), but the present data do not demonstrate an EP3-initiated signaling cascade. Whole-hypothalamus homogenates additionally average signals across anatomically and functionally distinct nuclei, limiting spatial attribution of the observed transcriptional changes.

### 4.5 CGA bioavailability, novelty, and study limitations

Orally consumed CGA can be absorbed as intact chlorogenic acids and related compounds, but extensive host and microbial metabolism can substantially alter the circulating metabolite profile (Farah et al., 2008; Gonthier et al., 2003). The present study did not quantify intact CGA, conjugated metabolites, microbial transformation products, or their concentrations in circulation or brain tissue. Consequently, hypothalamic transcriptional changes cannot be attributed specifically to direct exposure of the brain to intact CGA. Indirect effects through the intestinal environment, systemic mediators, or CGA-derived metabolites remain possible and should be tested directly.

Previous work from our group, including Veterinary Sciences 2022, 9:582 and subsequent studies, has already shown that CGA can influence broilers under stocking-density stress (Liu et al., 2023; Li et al., 2023; Bai et al., 2022). The additional contribution of the present study is the integrated evaluation of the four density × CGA treatment combinations together with jejunal microbial ecology, local SCFAs, and hypothalamic inflammatory- and appetite-related transcription.

Several limitations should be acknowledged. First, serum cytokines were evaluated as age-specific cross-sectional endpoints rather than as a repeated within-bird longitudinal panel, so temporal patterns are interpreted across sampling ages rather than as individual trajectories. Second, the study did not include cecal or circulating SCFAs, CGA pharmacokinetics, receptor-specific interventions, protein abundance or phosphorylation, or functional neural assays. Third, microbiota correlations remain associative. Finally, whole-hypothalamus qPCR does not resolve nucleus- or cell-specific responses. These limitations constrain causal interpretation but do not negate the coordinated treatment-associated patterns across growth, local intestinal ecology, metabolites, and hypothalamic transcription.

### 4.6 Implications and future directions

The present findings support CGA as a nutritional factor associated with selected responses under high stocking density, while the evidence does not justify describing a validated microbiota–gut–brain mechanism or a uniform density-specific rescue across endpoints. Future studies should extend the present factorial framework by pairing jejunal measurements with cecal and circulating or portal metabolite data, quantifying CGA and its metabolites, and incorporating protein/phosphorylation assays, receptor-specific interventions, and spatially resolved hypothalamic analyses. Such experiments would determine whether the observed intestinal and hypothalamic signals are causally connected or are parallel responses to the same nutritional and environmental intervention.

## 5 Conclusion

High stocking density was associated with poorer late-stage growth performance, age-dependent alterations in circulating cytokines, changes in jejunal microbial community structure, lower concentrations of several jejunal SCFAs, and altered hypothalamic inflammatory- and appetite-related gene expression. Dietary CGA was associated with selected growth, microbial, cytokine, and hypothalamic transcriptional responses. Jejunal propionate and butyrate showed numerical upward trends in HDCGA relative to HD, but the differences were not statistically significant. The coordinated microbial, metabolic, and hypothalamic transcriptional patterns are consistent with a potential microbiota–gut–brain association; however, the present data do not establish causal microbial–neural signaling or direct activation of GPR43/AMPK/NF-κB or EP3/MAPK pathways. Protein-level validation, receptor-specific perturbation, circulating metabolite measurements, and spatially resolved hypothalamic analyses are required to establish causality.

## Data Availability

The data presented in this study are deposited in the Genome Sequence Archive (GSA) at the National Genomics Data Center, China National Center for Bioinformation/Beijing Institute of Genomics, Chinese Academy of Sciences, under accession number CRA048503, and are publicly accessible at https://ngdc.cncb.ac.cn/gsa.
